# Co-inoculation of aflatoxigenic and non-aflatoxigenic strains of *Aspergillus flavus* to study fungal invasion, colonization, and competition in maize kernels

**DOI:** 10.3389/fmicb.2014.00122

**Published:** 2014-03-27

**Authors:** Zuzana Hruska, Kanniah Rajasekaran, Haibo Yao, Russell Kincaid, Dawn Darlington, Robert L. Brown, Deepak Bhatnagar, Thomas E. Cleveland

**Affiliations:** ^1^Geosystems Research Institute, Mississippi State University, Stennis Space CenterStarkville, MS, USA; ^2^Southern Regional Research Center, Agricultural Research Service – United States Department of AgricultureNew Orleans, LA, USA

**Keywords:** *Aspergillus flavus*, biocontrol, green fluorescent protein (GFP), fungal competition, aflatoxin, fluorescence microscopy, corn (*Zea mays*)

## Abstract

A currently utilized pre-harvest biocontrol method involves field inoculations with non-aflatoxigenic *Aspergillus flavus *strains, a tactic shown to strategically suppress native aflatoxin-producing strains and effectively decrease aflatoxin contamination in corn. The present *in situ *study focuses on tracking the invasion and colonization of an aflatoxigenic *A. flavus* strain (AF70), labeled with green fluorescent protein (GFP), in the presence of a non-aflatoxigenic *A. flavus* biocontrol strain (AF36), to better understand the competitive interaction between these two strains in seed tissue of corn (*Zea mays*). Corn kernels that had been co-inoculated with GFP-labeled AF70 and wild-type AF36 were cross-sectioned and observed under UV and blue light to determine the outcome of competition between these strains. After imaging, all kernels were analyzed for aflatoxin levels. There appeared to be a population difference between the co-inoculated AF70-GFP+AF36 and the individual AF70-GFP tests, both visually and with pixel count analysis. The GFP allowed us to observe that AF70-GFP inside the kernels was suppressed up to 82% when co-inoculated with AF36 indicating that AF36 inhibited progression of AF70-GFP. This was in agreement with images taken of whole kernels where AF36 exhibited a more robust external growth compared to AF70-GFP. The suppressed growth of AF70-GFP was reflected in a corresponding (upto 73%) suppression in aflatoxin levels. Our results indicate that the decrease in aflatoxin production correlated with population depression of the aflatoxigenic fungus by the biocontrol strain supporting the theory of competitive exclusion through robust propagation and fast colonization by the non-aflatoxigenic fungus.

## INTRODUCTION

The opportunistic, soil-borne fungus *Aspergillus flavus* is the main producer of aflatoxin, a mycotoxin known for its carcinogenic properties and other deleterious effects on health in both human and animal populations. Aflatoxins have been found in many agricultural commodities including corn, cottonseed, groundnut, and tree nuts (CAST, 2003), and contamination can occur at any time point between pre- and post- harvest; specifically, plant growth through crop storage. Aflatoxin affects food production and international commerce, prompting enforcement of regulations regarding acceptable aflatoxin limits in many countries around the world [Food and Agriculture Organization (FAO), 2004].

Different pre- and post-harvest strategies have been tested for the prevention and control of aflatoxin in order to protect the integrity of corn. Pre-harvest strategies include traditional and molecular breeding approaches to develop resistant germplasm (Rajasekaran et al., 2006; Cary et al., 2009), and biological control by utilizing various microorganisms including bacteria, yeasts, and non-aflatoxigenic fungi (Yin et al., 2008). The most promising pre-harvest biocontrol method involves field inoculation with non-aflatoxigenic *A. flavus*, a strategy that has proven successful at reducing aflatoxin contamination in the field (Cotty, 1994, 2001; Dorner, 2009a,b). Two bio-pesticides, AF36 and Afla-Guard®, have been approved by the Environmental Protection Agency (EPA) for pre-harvest application to control aflatoxin contamination in the U.S. (Moore et al., 2013). Neither AF36 (NRRL 18543) nor the component strain in Afla-Guard® (NRRL 21882) can produce aflatoxin due to genetic mutations in, or lack of, critical genes along the aflatoxin synthesizing pathway, respectively, (Ehrlich and Cotty, 2004; Chang et al., 2005; Moore et al., 2009). This method of control has been effectively tested on several commodities including cottonseed (Cotty, 1994, 2001), corn, (Brown et al., 1991; Dorner, 2009a), and peanuts (Dorner, 2009b). The suppression of measurable aflatoxin has been attributed to competitive exclusion of aflatoxigenic strains by non-aflatoxigenic strains; however, the exact mechanism of suppression has yet to be elucidated. Exclusion of the aflatoxin producing strain may be achieved by a more robust propagation and faster growth of the competing non-aflatoxigenic fungal strain (Chang and Hua, 2007). Alternatively, the inhibition of aflatoxin contamination/production could be due to a close physical proximity between the competing strains which may initiate a signal to down-regulate or inhibit synthesis of aflatoxin in the aflatoxigenic strain by the non-aflatoxigenic strain (Huang et al., 2011).

Dispersion of conidial inocula in the corn field is presumed to occur through a direct transfer by insects, or via colonization of silks, and subsequent movement toward the developing kernels (Payne, 1998; Mehl and Cotty, 2011). Entry into individual kernels is thought to occur primarily through the pedicel (tip cap) of intact kernels (Lillehoj, 1983; Rajasekaran et al., 2013). However, it is difficult to determine the mode of action taken by the competing fungal strains, particularly during the infection and colonization process inside individual kernels.

The use of fungal strains that express fluorescent proteins in the cytoplasm greatly enhances the potential for visual tracking of biological processes without markedly affecting fungal growth or pathogenicity (Rajasekaran et al., 2008; Crespo-Sempere et al., 2011). Enhanced forms of the green fluorescent protein (eGFP) were recently developed to tag *A. flavus* for study of fungal growth, mode of entry and colonization of cottonseeds (Rajasekaran et al., 2008), to track the progress of fungal infection within developing corn ears (Magbanua et al., 2013), to study infection, fungal growth, colonization, and aflatoxin production in intact corn kernels (Rajasekaran et al., 2013), and also to tag *A. carbonarius* for monitoring fungal colonization in grapes (Crespo-Sempere et al., 2011).

To better understand the mechanisms involved during competition between co-inoculated *A. flavus* isolates in corn, the current *in situ* study focuses on tracking the invasion and colonization by aflatoxigenic AF70, labeled with GFP, in the presence of the AF36 biocontrol strain.

## MATERIALS AND METHODS

### FUNGAL STRAINS AND PREPARATION OF INOCULA

Two *A. flavus* strains were used as competitors. Toxin producing AF70 labeled with GFP (AF70-GFP; Rajasekaran et al., 2008), and a non-aflatoxigenic strain of *A. flavus* (AF36) fungal cultures were obtained from the SRRC fungal collection (ARS-USDA, New Orleans, LA, USA), and grown on potato dextrose agar (PDA) media for 7 days at 30°C. Bright green fluorescence was observed in the AF70-GFP culture from both the conidiophores and the mycelia. Harvest of conidia was accomplished by flooding a single culture for each strain with 20 ml of 0.01% (v/v) sterile Triton X-100 solution and scraping the surface mycelia with a sterile scraper. Conidial suspensions were adjusted to 4 × 10^6^ cells/ml using sterile distilled water. The inocula were kept in separate containers at 4°C. Immediately before use, 50 ml of each inoculum, at 1:1 ratio, was combined to make up the AF70-GFP+AF36 co-inoculation mixture.

### *IN SITU* INOCULATION

**FIGURE 1:**
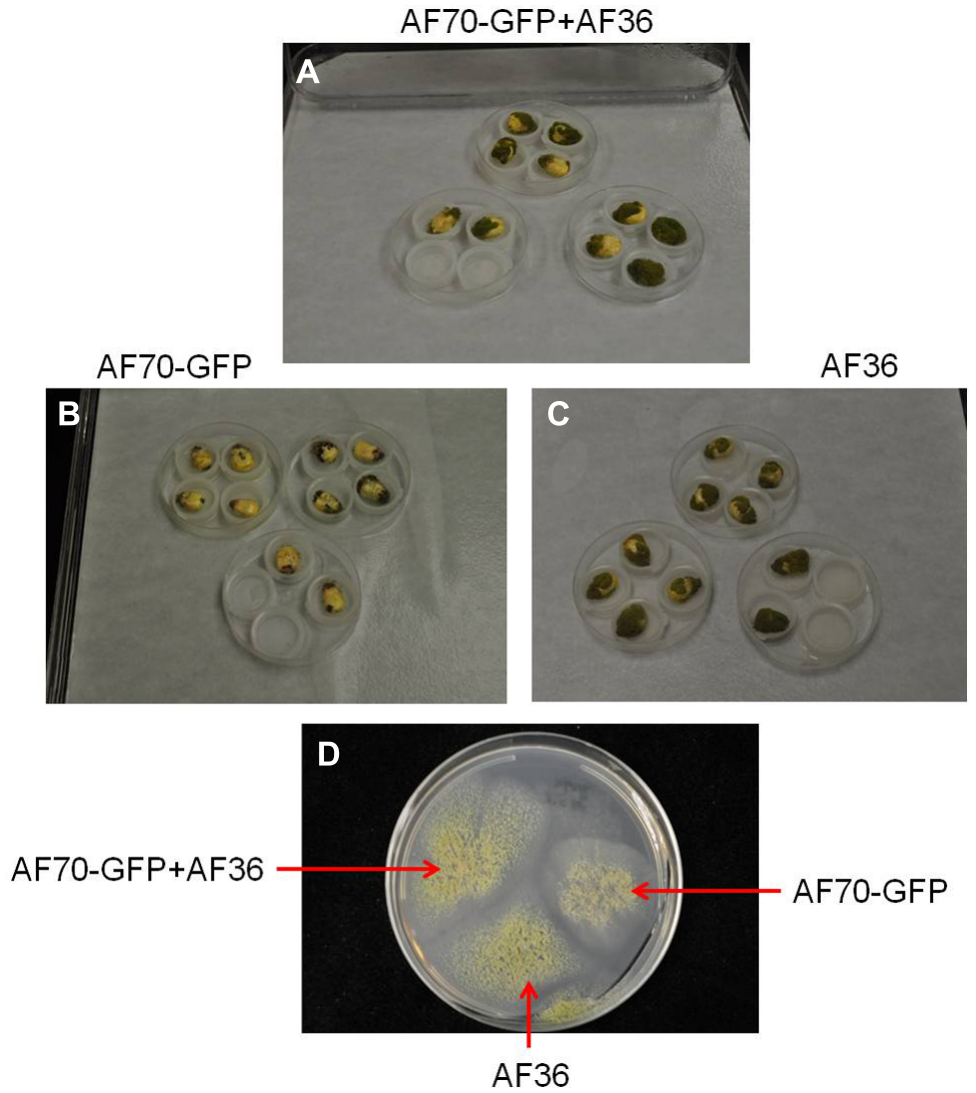
**Images of *A. flavus* fungal isolates AF70-GFP and AF36 in a mixture **(A)** or grown separately **(B,C)** on inoculated corn kernels and in culture **(D)**; compared on the seventh day of growth**.

Corn kernels (N78B-GT, Syngenta NK Brand Seeds, Laurinburg, NC, USA), collected in 2010 from the ARS Field Station in Stoneville, MS, USA, were utilized in all experiments. Whole, undamaged kernels of roughly uniform size were randomly assigned into four treatment groups and processed according to a modified kernel screening assay (KSA; Brown et al., 1995). All kernels were surface sterilized in 70% ethanol and rinsed in dH_2_O. Ten kernels per treatment per day were inoculated by immersion and stirred for 1 min. The treatments included:(1) kernels co-inoculated with AF70-GFP+AF36, (2) kernels inoculated with AF70-GFP only, (3) kernels inoculated with AF36 only, and (4) kernels inoculated only with dH_2_O as control for non-specific fluorescence, and/or inherent fungal contamination. Each group of kernels was incubated in a humidity chamber using a plastic tray with individual compartments (**Figure 1**). Kernels were incubated at 30°C and examined at several time points (3, 4, 5, 7, and 9 days) after inoculation.

#### GFP imaging and analysis

At each specified time interval, kernels were removed from the incubator, wiped free of visible exterior mold growth, sliced longitudinally, and mounted on a slide with double sided tape. A total of 20 kernel cross-sections per treatment per day were analyzed for GFP fluorescence. Images were taken in the dark with an Olympus SZH10 GFP stereomicroscope (Olympus, Center Valley, PA, USA) equipped with 480 nm excitation and 535 nm emission filters for GFP fluorescence, and also with UV filters. Digital images were acquired with a Nikon Digital Camera DXM1200 (Nikon Instruments, Melville, NY, USA). Sectioned samples were then processed for aflatoxin analysis.

In order to determine the extent of suppressed expression of the GFP signal from the AF70-GFP by AF36 in the co-inoculated corn kernels, as compared with those kernels inoculated with AF70-GFP alone, all GFP-expressing images were analyzed with pixel count analysis in ENVI (ENVI software v 4.7, ITT Exelis, Boulder, CO, USA). Pixel count analysis has been employed previously as a useful tool for quantifying fluorescence signals in digital images (Waters, 2009). Each 8-bit image contained 3200 x 2560 pixels with brightness values ranging from 0–255. In order to quantify the number of pixels exhibiting fluorescence within each image, a region of interest (ROI) was created by utilizing a band thresholding process. A threshold brightness value of 20 was selected to separate the pixels exhibiting fluorescence (>20) from the background (<20) and minimize non-specific background fluorescence. Regions of interest were drawn around all pixels exhibiting values above the set threshold, and totals for each cross section of each sample were recorded for statistical analysis.

### AFLATOXIN ANALYSIS

Imaged kernels were dried at 60°C for 2 days and analyzed for aflatoxin with the VICAM AflaTest assay (VICAM, Milford, MA, USA). Dried kernels were processed following the single kernel assay protocol of Yao et al. (2010), modified from the VICAM AflaTest instruction manual for corn, milo, grains, and feeds. Briefly, kernels from each day were first uniformly, semi-coarsely ground in a coffee grinder (KitchenAid® BCG100). Next, aflatoxin B from each ground sample was extracted with sodium chloride (NaCl) in methanol:water (80:20 v/v) maintaining the proportion of 1 g of sample plus 0.1 g NaCl in 2 ml of methanol:water, and filtered through a fluted filter. Filtered extract was diluted 1:5 with ultrapure water; 2 ml of each sample were passed through a glass filter and pushed through the high affinity AflaTest column. Samples were washed with ultrapure water, eluted with pure methanol (HPLC grade), and fluorescence was measured using the VICAM Series-4EX fluorometer (VICAM, Milford, MA, USA). Raw data values were expressed as ppb or μg/kg equivalents.

### STATISTICAL ANALYSES

Pixel counts from the images, as well as the aflatoxin concentrations from the aflatoxin extractions, were analyzed in a two-way ANOVA with repeated measures using the Excel Data Analysis package (Excel 2007, Microsoft Office, Microsoft). Graphs were created in the R statistical program (R statistics software, http://www.r-project.org).

## RESULTS

### GFP IMAGING AND POPULATION (PIXEL) COUNT

**FIGURE 2:**
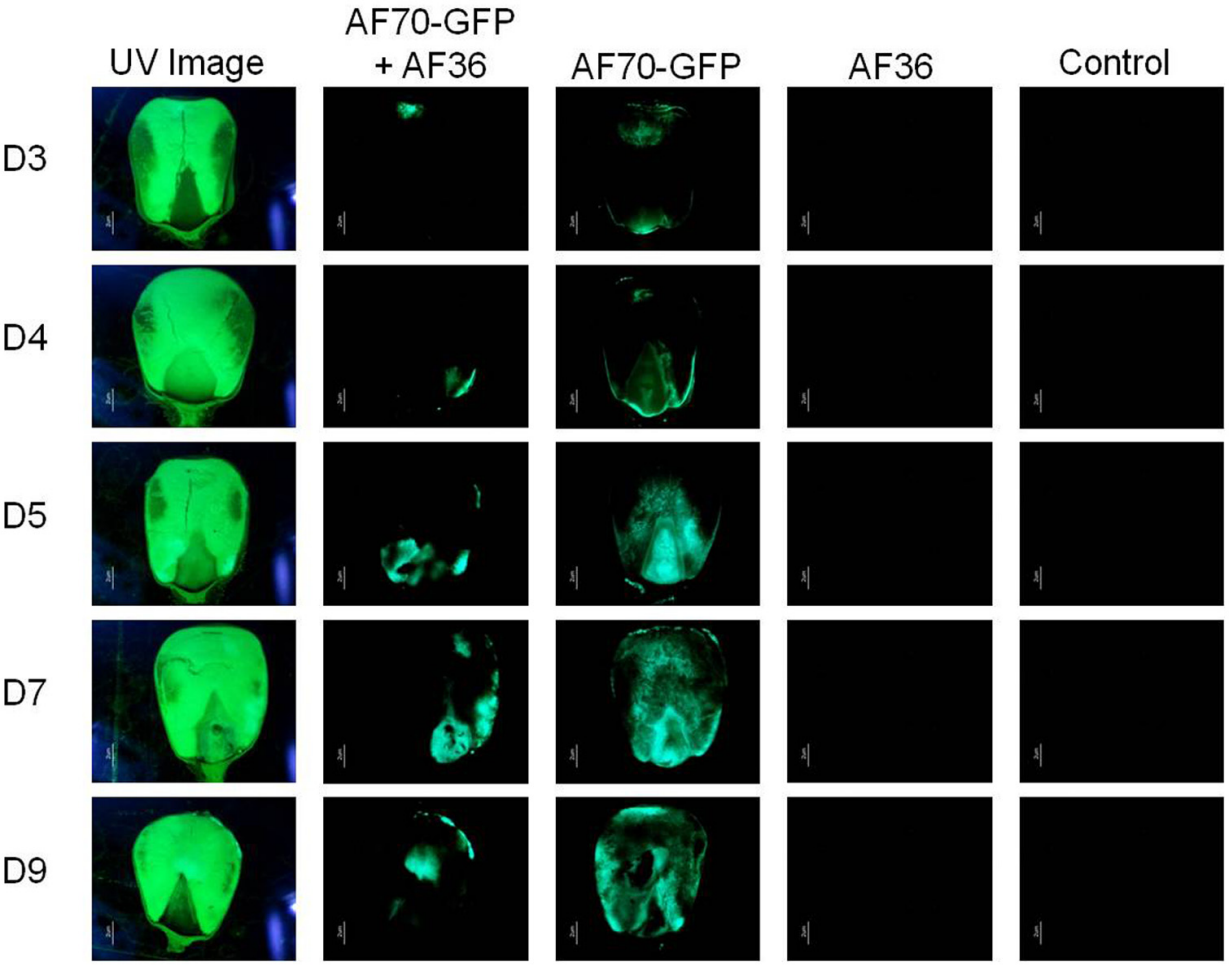
**Fluorescence images representing inoculation treatments observed using UV light (first column) and using a GFP filter (columns two through five) for each day of examination (day 3–day 9; D3–D9).** Columns one and two show co-inoculated kernels of AF70-GFP+AF36; columns three through five shows kernels inoculated with AF70-GFP alone, AF36 alone, and a negative control using sterile water, respectively.

On intact corn kernels the observed external fungal growth was predominantly AF36 for the co-inoculated groups; in some instances AF36 completely covered the pericarp (**Figure 1**). Internally, the spread of AF70-GFP was evident by the presence of fluorescence (**Figure 2**). In corn kernels inoculated solely with AF70-GFP, the fungus spread unimpeded, filling the whole kernel from the basal transfer layer through the embryo and into the endosperm, eventually compromising the kernel’s integrity. Although the point of entry was predominantly at the pedicel, it was not unusual to find GFP fluorescence in other areas of a kernel appearing first. The co-inoculated kernels exhibited a radically different fungal spread (**Figure 2**). The AF70-GFP growth appeared limited to the edges of the corn kernel, presumably outcompeted by AF36. For kernels inoculated with AF36 alone, or with the sterile water control, there was no GFP fluorescence observed; therefore, they were not included in the pixel count analysis. No GFP fluorescence could be observed externally on co-inoculated kernels which supports the premise that AF36 predominated.

**FIGURE 3:**
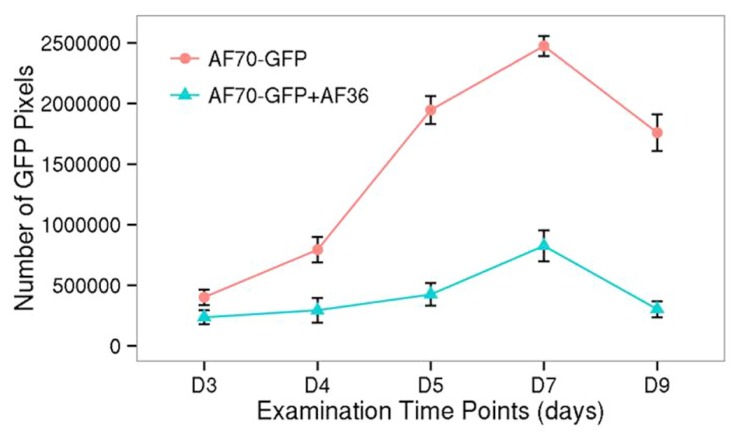
**GFP fluorescence at each time point (day 3–day 9 after inoculation), collected as pixel counts, from images of corn kernels inoculated with AF70-GFP+AF36 or the AF70-GFP isolate alone.** The AF70-GFP+AF36 mixture is represented by the green line and AF70-GFP alone is represented by the red line. There was a significant treatment effect *p* <0.001, and a statistically significant interaction of treatment by day *p* <0.001, α = 0.01.

**FIGURE 4:**
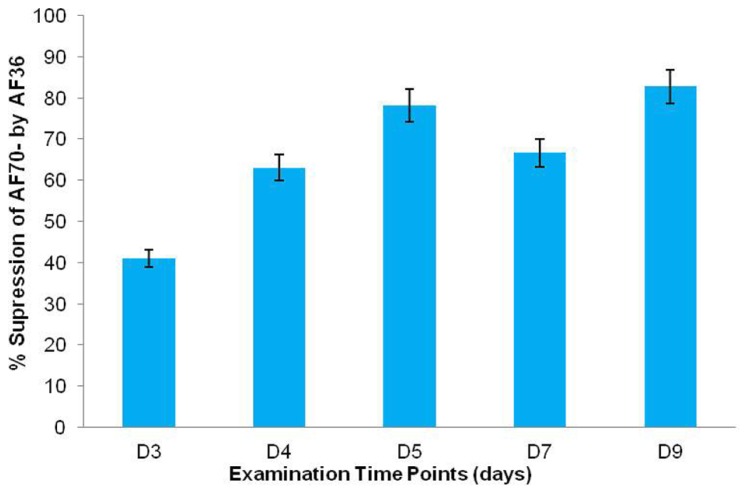
**Percent (%) growth suppression of AF70-GFP by AF36 in co-inoculated corn kernels as determined by calculating the percent difference between treatment means on a particular day.** Over 5% difference in suppression was observed between consecutive time points (day 3–day 9).

Statistical analysis of the fluorescent pixels occupied by AF70-GFP is presented in **Figure 3**. Data was analyzed in a two way ANOVA using each treatment (AF70-GFP+AF36 and AF70-GFP) with repeated measures of day (post-inoculation). There was a significant main effect of treatment with *p* <0.001, and a statistically significant interaction of treatment by day *p *<0.001, α = 0.01. These results indicate that there was a significant difference between co-inoculated kernels and those treated with AF70-GFP alone, and this difference changed over time. Suppression of AF70 by AF36 is presented in terms of percentages in **Figure 4**. The peak difference between the two groups appeared to be on day nine whereby the highest suppression of AF70-GFP by AF36 was observed to be 82%. Average suppression over the duration of the study was 66%.

### AFLATOXIN CONTENT

**FIGURE 5:**
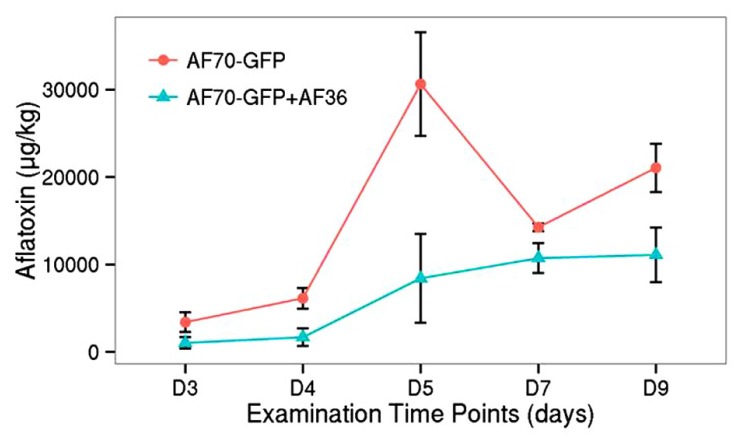
**Aflatoxin concentration for co-inoculated corn kernels treated with AF70-GFP+AF36 (green) or AF70-GFP (red) alone from day 3 to day 9 after inoculation.** There was a significant treatment effect *p* <0.001, and a statistically significant interaction of treatment by day *p* <0.001 with α = 0.01.

Similar to the population count, the aflatoxin data was analyzed in a two way factorial design of each treatment (AF70-GFP+AF36 and AF70-GFP) with repeated measures of day (**Figure 5**). A significant main effect of treatment with *p* <0.001, and a statistically significant interaction of treatment by day *p* <0.001 with α = 0.01 were revealed, indicating a significant difference in aflatoxin concentration between the co-inoculated kernels and those treated with AF70-GFP alone that changed over time. The peak difference between the two groups appeared to be on day five (73%), dipped by day seven (25%), and recovered to a lesser degree on day nine (47%). The observed suppression of AF70-GFP by AF36 presented in **Figure 4** is also observed in the aflatoxin content data presented in **Figure 5**. Maximum decrease in aflatoxin production was determined by calculating the percent difference between means on a particular day and was 73% on day five.

## DISCUSSION

The current study used an *A. flavus* strain, expressing the GFP gene, to investigate its invasion and colonization potential in the presence of a non-aflatoxigenic biocontrol strain in single corn kernels. Although all co-inoculated corn kernels exhibited growth on day three, external fungal growth over the entire growth period was predominantly attributed to AF36, confirmed by lack of fluorescence combined with aggressive growth. Furthermore, the fluorescence was very specific to AF70-GFP and was not present externally or internally in the AF36 controls. It appears the competitive edge exhibited by AF36 may be in its propensity for rapid growth and aggressive colonization of the host compared to AF70-GFP (**Figure 1**). Aflatoxin production requires expending energy in the form of ATP (Ehrlich et al., 2011), and perhaps this affects a toxigenic strain’s ability to rapidly colonize, since it may be diverting energy toward the secondary metabolism pathway. Because this does not appear to be an effective competitive strategy targeted against non-aflatoxigenic strains, it appears that aflatoxigenic and non-aflatoxigenic strains are not in competition with each other, under natural conditions, and may coexist without interfering with each other’s life cycles unless their natural proportions are disturbed, for example, by inundating fields with excess biocontrol (Yin et al., 2008).

Although the initial entry of the fungus through the pedicel agrees with previous research (Rajasekaran et al., 2013), there were instances where the GFP fluorescence initially appeared in other areas of the corn kernel, particularly when AF70-GFP and AF36 were co-inoculated. The AF70-GFP fluorescence in the co-inoculated kernels was significantly reduced and limited to the edges of the kernels, presumably because of the more aggressive growth of the AF36 biocontrol strain. Entry points were randomly distributed along the hull, possibly created by peripheral damage to the pericarp by the robust growth of AF36 where the extensive hyphal growth may have weakened the external kernel tissue. Alternately, invisible weak points in the external tissue may have been created during kernel harvest. Greatest population difference observed for GFP fluorescence between the AF70-GFP and AF70-GFP+AF36 treatments are in agreement with the literature, whereby population, aflatoxin production was decreased by aflatoxin non-producers between 70 and 90% (Brown et al., 1991). The average suppression of aflatoxin production in the current study was 50% compared to results reported in previous studies involving cotton (Cotty, 1990, 1994). The discrepancy may be attributed to the shorter duration and a smaller sample size of the laboratory experiment, compared to field experiments. The fact that AF36 was first isolated from cotton and may have a host preference could be another reason for the difference. The timing of the inoculation may have contributed to the reduced inhibition of aflatoxin production observed in this study. Greater reduction (80–90%) of aflatoxin concentration in corn (Brown et al., 1991), and cottonseed (Cotty, 1990), was shown when the non-aflatoxigenic fungal strain was introduced 24 h prior to the aflatoxin producing strain rather than simultaneously. However, this may not be a reflection of what is actually happening in the field. Studies that explore pre-inoculation with aflatoxigenic strains would better demonstrate the field environment.

The observed population differences between the co-inoculated and the sole AF70-GFP treatments support the premise that growth suppression of AF70-GFP is due to competitive exclusion by AF36 (Brown et al., 1991; Mehl and Cotty, 2010). The exclusion could be due to the initial acquisition of nutrients or tissue space by the biocontrol strain, or something unknown that the fungus requires for survival in a particular situation (Mehl and Cotty, 2011, 2013). Since spore concentrations for inocula in our competitive study were approximately the same, faster growth, and a more robust propagation of AF36 effectively excludes the aflatoxin producing strain and consequently decreases measurable aflatoxin levels. An additional possibility for decrease in aflatoxin production is presented by the presence of specific volatiles. Aflatoxigenic fungi reportedly produce volatile compounds that differ from non-aflatoxigenic fungi (De Lucca et al., 2012). Certain terpenes found in essential oils, such as alpha-pinene have known antifungal properties (Moghtader et al., 2011) and have been discovered among the volatile compounds produced by some non-aflatoxigenic *A. flavus* isolates (De Lucca et al., 2012). Furans are another class of anti-microbial volatiles produced by *A. flavus*; however, in non-aflatoxigenic strains they are released several days earlier than in aflatoxin producing strains (De Lucca et al., 2012). A possible competitive strategy may involve the production of specific volatiles by the non-aflatoxigenic fungal strains to inhibit growth progression of the aflatoxin producers which would affect the population size of the aflatoxigenic fungi, and consequently reduce the amount of aflatoxin produced. Because the non-aflatoxigenic strains do not appear affected by close proximity to aflatoxin producers, it seems unlikely non-aflatoxigenic strains target aflatoxin production specifically. Therefore, the decrease in aflatoxin may be an indirect result of the suppressed population size of the aflatoxin-producing strain.

This strategy would help explain the apparent success shown by using non-aflatoxigenic fungi to effectively control aflatoxin contamination under field conditions. Unfortunately the long-term consequences of this form of treatment have not been studied. Our study has demonstrated that although AF36 decreased aflatoxin production, the kernels colonized by the biocontrol strain were severely damaged by day nine. There may not have been aflatoxin produced by the AF36 fungus; however, by the end of the study most of the kernels lost their integrity and were overwhelmed by the fungus which may not benefit the crop should the biocontrol strain be allowed to remain during post-harvest storage. Another potential caveat to use AF36 as biocontrol is that it produces cyclopiazonic acid (CPA) which is a mycotoxin that reportedly targets some internal organs as well as the skeletal muscles (Chang et al., 2009; Abbas et al., 2011; Moore et al., 2013). Although the toxic effects of CPA are not as well-documented as the effects of aflatoxin (Chang et al., 2009), overuse of the *A. flavus* isolates that produce CPA may lead to unintended effects on animal and human health. Additionally, when using non-aflatoxigenic fungal strains for applications in biocontrol, it is also important to take into account the potential course the suppressed aflatoxigenic strains may take to overcome inundation and suppression by the competing non-aflatoxigenic strains. For example, the constantly suppressed strain may call upon its latent ability for sexual reproduction demonstrated under laboratory conditions resulting in generations of aflatoxigenic offspring far more virulent than anticipated (Moore et al., 2013). All of these concerns regarding potential effects on crop integrity and/or unanticipated health risks need to be carefully evaluated when considering specific non-aflatoxigenic strains for aflatoxin biocontrol applications.

The present study has offered *in situ* evidence that the AF36 biocontrol strain is successful at suppressing tissue proliferation, and subsequently, aflatoxin contamination by an aflatoxigenic *A. flavus* isolate. Use of a GFP-labeled, aflatoxin-producing isolate allowed us to easily track invasion and colonization for a better understanding of the competitive relationship between the two strains in corn kernels. Our findings support the theory of competitive exclusion, in favor of the biocontrol strain, based on its robust growth and proliferation. However, this study also points to valid concerns regarding the long-term use of non-aflatoxigenic fungi for suppression of native toxigenic strains in biocontrol strategies.

## Conflict of Interest Statement

The authors declare that the research was conducted in the absence of any commercial or financial relationships that could be construed as a potential conflict of interest.
